# A Case of a Successfully Treated Patient With a Delayed Diagnosis of Boerhaave’s Syndrome and Severe Complications

**DOI:** 10.7759/cureus.74018

**Published:** 2024-11-19

**Authors:** Georgi Yankov, Magdalena Alexieva, Zaharinka Makshutova, Borislav Vladimirov, Mila Kovacheva-Slavova

**Affiliations:** 1 Thoracic Surgery, University Hospital “St. Ivan Rilski”, Sofia, BGR; 2 Gastroenterology, Institute for Specialization and Mastering of Doctors, University Hospital Tsaritsa Ioanna, Sofia, BGR

**Keywords:** boerhaave's syndrome, empyema, esophageal rupture, esophagectomy, mediastinitis, treatment

## Abstract

Boerhaave's syndrome is a rare critical condition manifesting as transmural esophageal rupture. It is usually associated with forceful emesis and increased intraesophageal pressure. Immediate aggressive surgical intervention is imperative in such cases. We present a patient with a late diagnosis of Boerhaave's syndrome who was successfully treated in our department. We performed a subtotal esophageal resection, esophagostomy, gastrostomy, pyloroplasty by Heineke-Mikulicz, debridement, bilateral early decortication of the lung, and cleaning of the pleural cavities and mediastinum.

## Introduction

Boerhaave's syndrome is a rare, critical condition manifesting as transmural esophageal rupture. It is usually associated with forceful emesis and increased intraesophageal pressure. Of all esophageal perforations, 10%-15% are due to this condition [1-3]. The diagnosis of this syndrome might be very difficult and often leads to delays. To diagnose Boerhaave's syndrome, a computed tomography (CT) scan or an esophagography with water-soluble contrast are appropriate methods as endoscopy is controversial because of the possibility of the rupture expanding. Mortality rates are up to 60% after intervention and up to 100% if it is not accomplished [4]. Treatment approaches are strictly individualized for each patient.

## Case presentation

**Figure 1:**
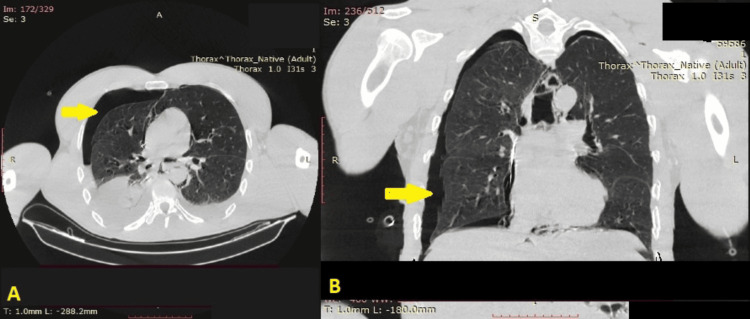
A) Axial and B) coronal CT views of bilateral hydropneumothorax and mediastinal emphysema (arrows) CT: computed tomography

**Figure 2:**
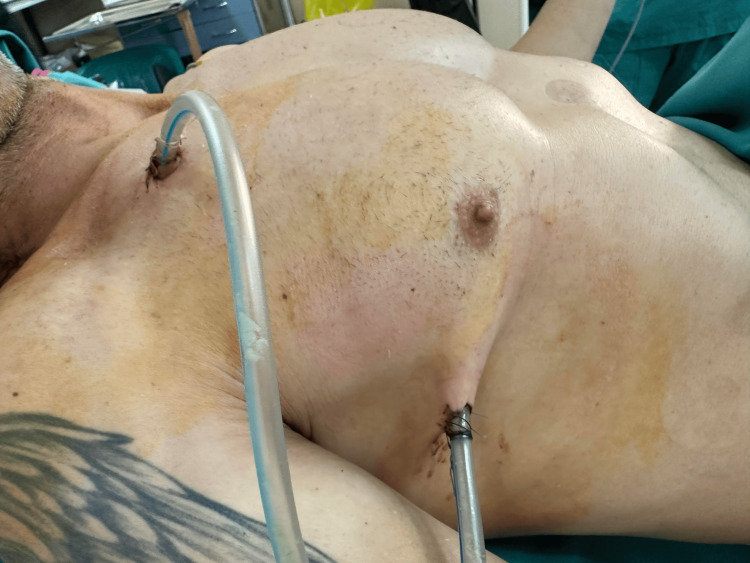
Preoperative view of the patient with two pleural catheters on admission

A 47-year-old man presented six days prior to his admission to our department with continuous vomiting and epigastric pain. As his overall condition worsened in the next 24 hours, he was hospitalized in another hospital and was diagnosed with a hydropneumothorax on X-ray. A coronary event was ruled out. In the cardiac surgery department of the hospital, the first thoracic catheter was placed in the mid-axillary line at the level of the sixth intercostal space, and about two liters of unclear fluid and air were evacuated. The patient was immediately referred to the general surgery department with a suspected pyopneumothorax. A native CT scan showed a total pneumothorax on the right with fluid levels, the presence of a thoracic catheter, pneumomediastinum, and a partial apical pneumothorax on the left side (Figure 1A, 1B). The doctors in this department urgently placed another thoracic catheter in the second intercostal space along the midclavicular line, which is an uncommon practice, rarely performed in cases of apical empyema collections, apical pneumothoraces, post-lung resections, and apical symptomatic residual cavities. Three days later, the patient's condition began to deteriorate esophageal contents and nutrients began to leak through the drains and after consultations with gastroenterologists, the patient was referred to our thoracic surgery department in severe septic condition. As esophageal rupture was highly suspected, a slightly diluted solution of methylene blue was given orally to the patient, the latter appeared through the drains, and the diagnosis of esophageal rupture was confirmed. The patient was referred for emergency surgery (Figure 2). 

**Figure 3:**
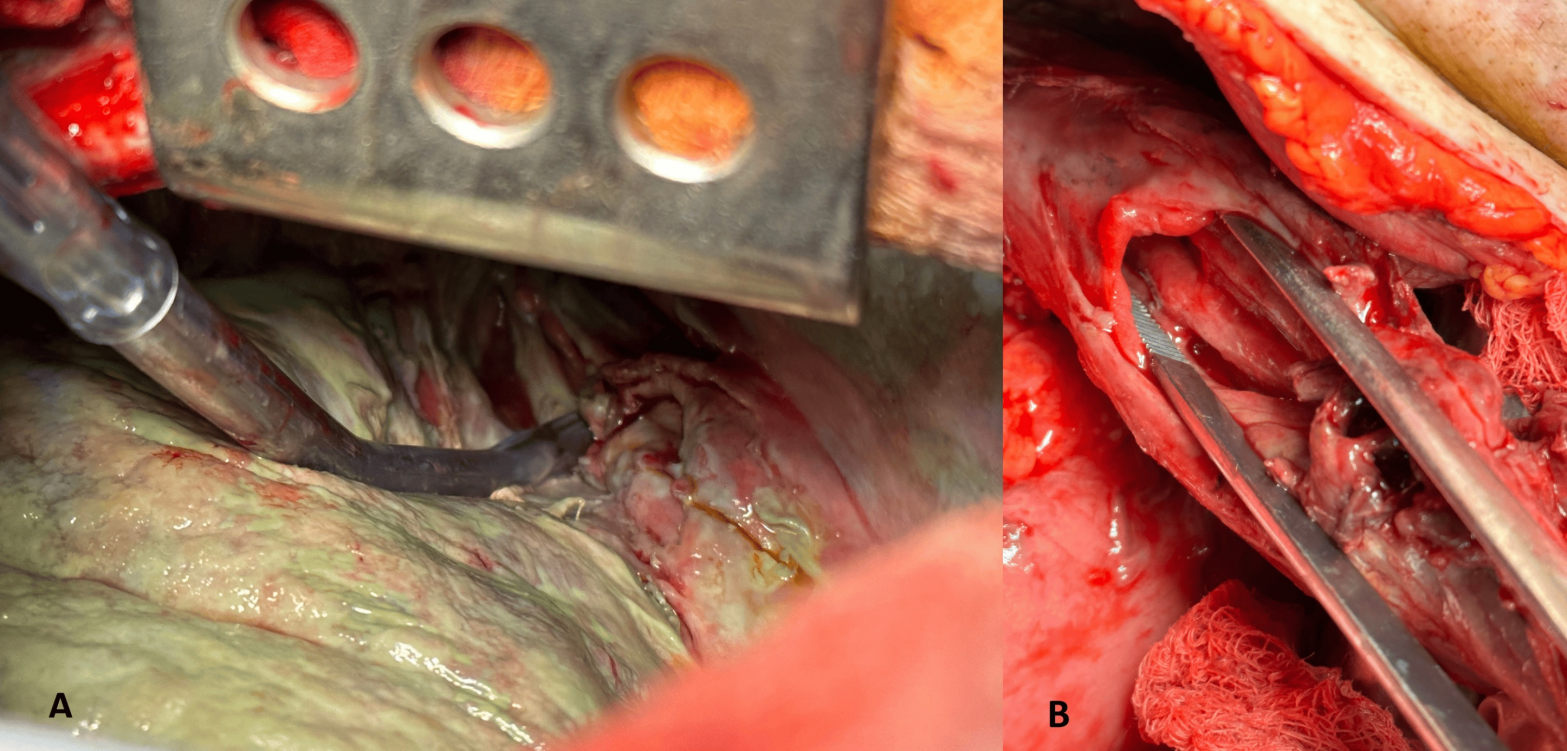
A) Intraoperative view showing the rupture of the esophagus, along with thickened and inflamed mediastinal, and visceral pleura of the right lung. B) Intraoperative view of the esophageal rupture, with a tweezer inserted into it

A right posterolateral thoracotomy was performed, with the removal of the inserted catheters prior to the operation. We observed purulent exudate in the right pleural cavity with loculations in the basal part, which delocalized the thickened visceral, costal, mediastinal, and diaphragmatic pleura, and an acute purulent empyema (Figure 3). We evacuated circa 500 mL of purulent exudate. In the distal third of the esophagus, along its right lateral wall and reaching almost to the cardia, we found a rupture approximately 5 cm in length and about 2 cm in width, with everted and smoothed mucosal edges (Figure 3).

**Figure 4:**
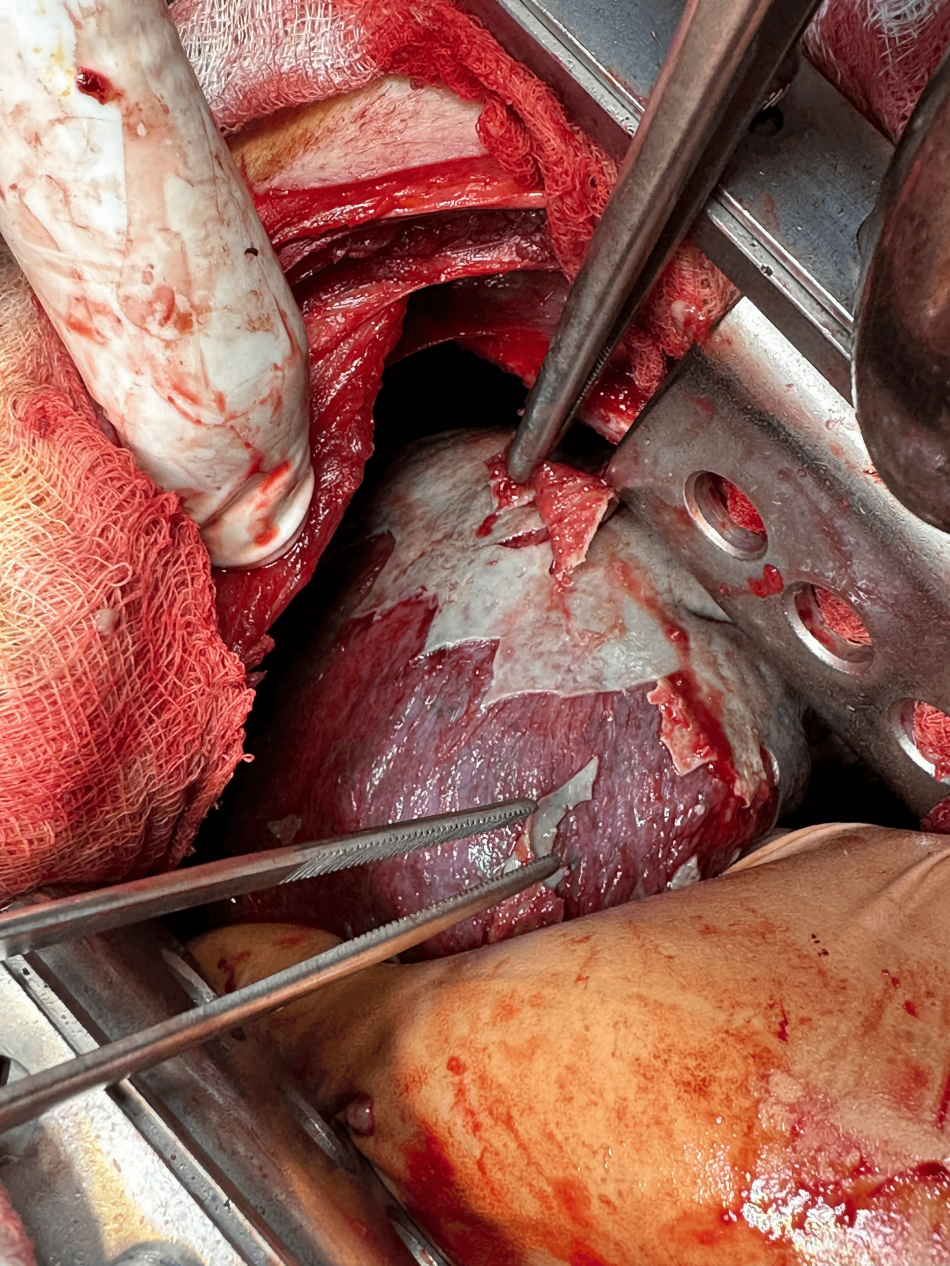
Intraoperative view of the decortication of the right lung with thickened visceral pleura

We incised the mediastinal pleura from the upper thoracic aperture to the hiatus. The presence of acute purulent mediastinitis was established. After debridement, purulent material was evacuated, and the thoracic esophagus was mobilized through blunt and sharp dissection (Figure 4), followed by a repeated lavage of the pleural cavity and mediastinum with diluted Braunol solution.

**Figure 5:**
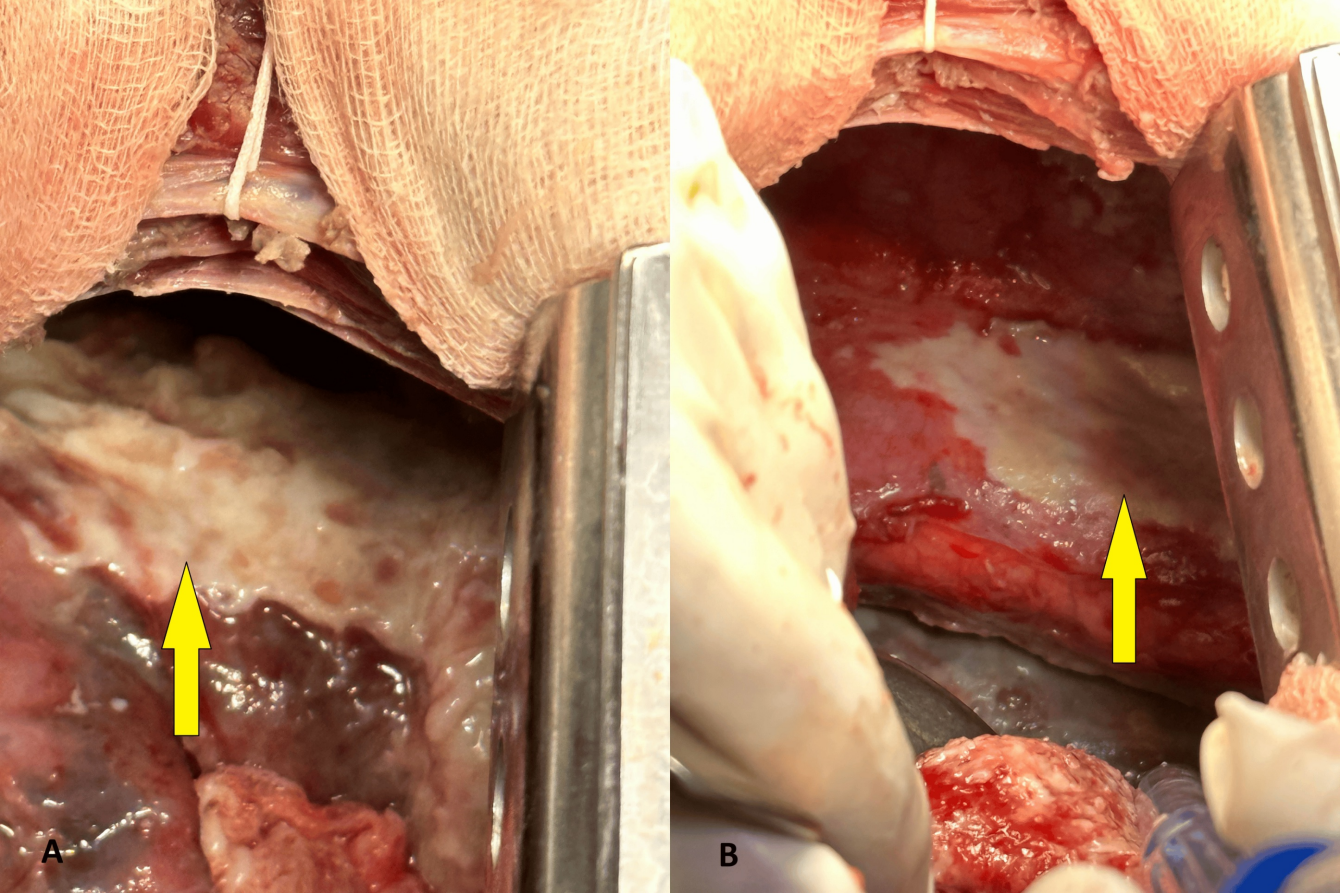
A and B show an intraoperative view of the left side with fibrin deposits (arrows), opacified visceral, and parietal pleura

**Figure 6:**
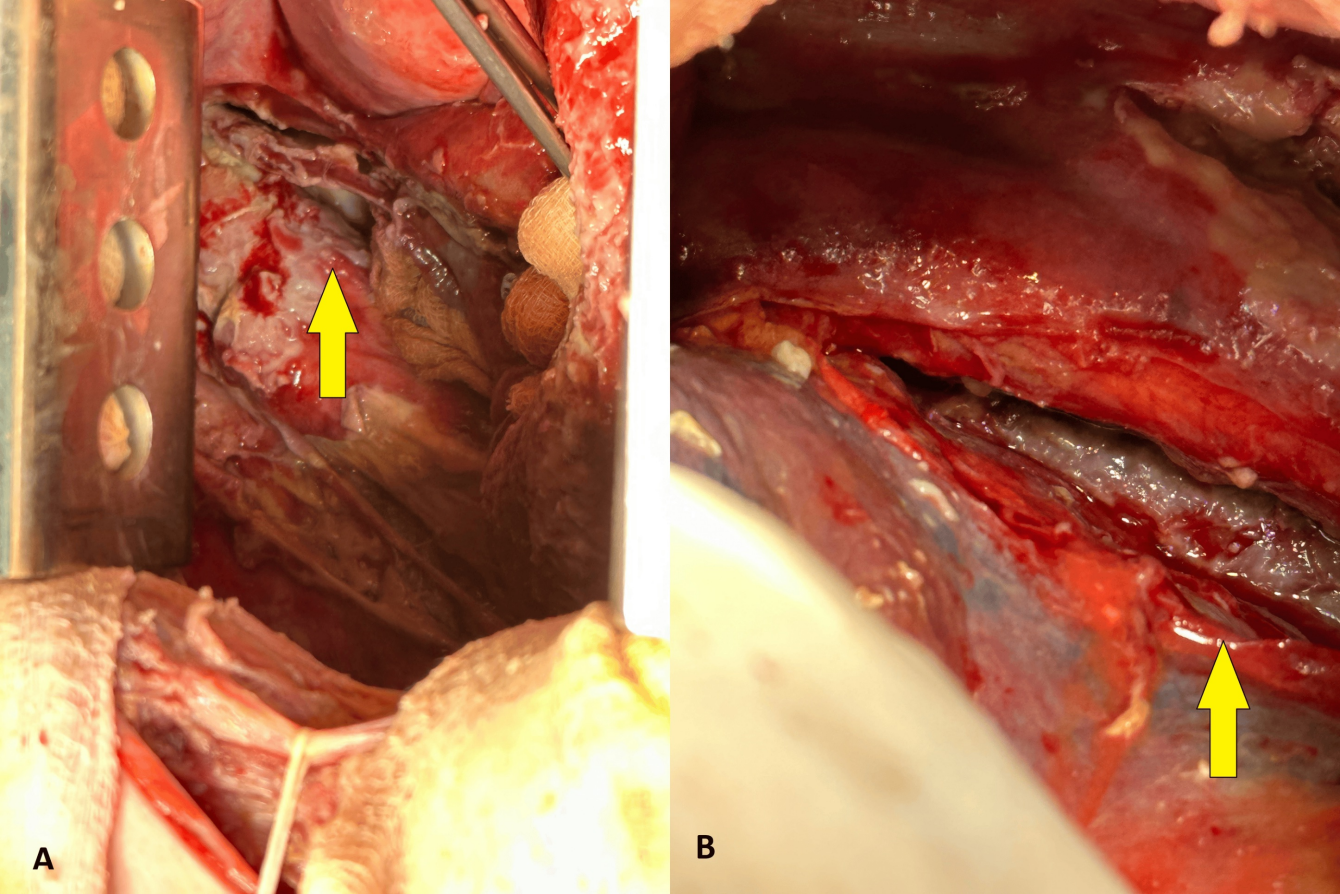
A and B show intraoperative views of the mediastinum and left pleural cavity after debridement and incision of the mediastinal pleura through a left thoracotomy approach

**Figure 7:**
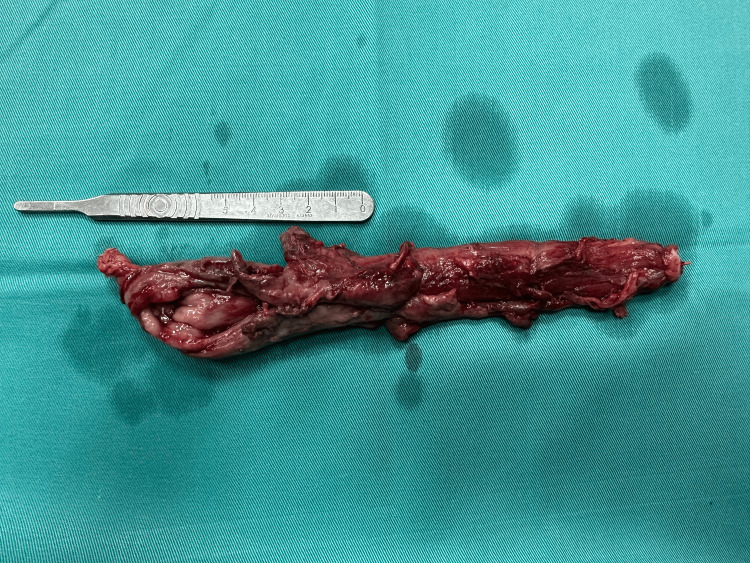
A postoperative specimen of the resected esophagus with the rupture site

We placed two catheters for active aspiration. After closing the right thoracotomy, we proceeded with a simultaneous left cervicotomy in front of the left sternocleidomastoid muscle and an upper median laparotomy. The esophagus was resected approximately 3 cm below the hypopharynx and its oral end was brought into a terminal esophagostomy. We performed a subtotal esophagectomy with transection of the esophagus in the gastroesophageal junction. There was a dense area in the pyloro-duodenal region, resembling an old cicatrized ulcer. A Heineke-Mikulicz pyloroplasty was performed. Through a separate left vertical transrectal incision, we performed a gastrostomy, followed by a closure of both laparotomy and cervicotomy. The position of the patient changed and а left lateral muscle-sparing minithoracotomy was performed. We found a purulent exudate in the left pleural cavity with dorsobasal loculations. Debridement was performed, and 500 mL of purulent fluid was evacuated. Fibrin deposits were present on the visceral, costal, and mediastinal pleura (Figure 5A, 5B). A mediastinal pleura was incised from the upper thoracic aperture to the hiatus and both the anterior and posterior mediastinum were opened (Figure 6A, 6B). Debridement and decortication with repeated lavage of the pleural cavity and mediastinum with diluted Braunol solution followed. We placed two catheters on active aspiration in the left pleural cavity. The left thoracotomy was closed. The macroscopic view of the resected esophagus is shown in Figure 7.

The histological result showed an esophageal wall with focal superficial ulceration and proliferation of granulation tissue, reactive changes with inflammatory infiltrates, and a fibrinous-purulent inflammatory process in the adventitia. Two lymph nodes showed reactive non-specific changes. The costal and mediastinal pleura exhibited fibrinous-purulent pleurisy.

The postoperative period was severe with daily increases in body temperature up to 38.5, which gradually decreased to normal by the 10th postoperative day. Тhe patient was discharged on the 17th postoperative day. Four months later he is in excellent general condition and will be prepared for reconstruction of the digestive tract. 

## Discussion

The first description of Boerhaave's syndrome dates back to 1724 by Hermann Boerhaave, who described the symptoms and death of an admiral of the Dutch fleet after profuse vomiting [2]. Mackler's triad is rarely encountered in Boerhaave's syndrome, but the most frequent symptoms in patients with hydropneumothorax are vomiting, chest pain, and dyspnea. For the successful treatment of this life-threatening condition, early and correct diagnosis, along with the presence or absence of an underlying disease such as motility disorders or benign and malignant esophageal strictures, is of paramount importance. Treatment options range from conservative approaches to major surgical interventions [5-7]. Conservative treatment methods include drains of the pleural cavities alone or in combination with endoscopic minimally invasive techniques. In case of early detected and small ruptures, it is possible to use conservative endoscopic techniques by means of clipping and placing a fully covered self-expanding metallic stent, or endoscopic ligation with snare loops [3,8,9]. Another option is the endoscopic placement of an open-pore polyurethane foam or luminal sponge [8]. If the diagnosis is made within the first 24 hours, the prognosis is better and the mortality is lower [5]. Surgical options are esophageal suture and reinforcement with vital tissue such as intercostal muscle, diaphragmatic, pleural, and pericardial flaps. Defect closure with interrupted stitches and the creation of an omental patch is a safe method for buttressing the suture line [10,11]. In such cases, additional laparoscopy or laparotomy is required to mobilize the omentum. Other options are placement of a T-tube drainаge, exclusion and divеrsion with a cervical stoma, and esophageal resection with reconstruction of the digestive tract at the same stage or after the patient's recovery. Minimally invasive techniques such as video-assisted thoracoscopic surgery (VATS) can also be considered for hemodynamically stable patients and early ruptures [10]. Ruptures in the distal intra-abdominal part of the esophagus and a clinical presentation of an acute surgical abdomen could also be treated with laparoscopic techniques. A case of resection and reconstruction of the esophagus in two stages over three days is also described [6].

A surgical or conservative approach in patients diagnosed after 72 hours remains debatable, despite surgery being the most effective method for treatment [12,13]. In the presented case, we were able to treat a patient successfully with a delayed spontaneous rupture of the esophagus with severe mediastinitis and bilateral empyemas. In reality, the initial clinical manifestations were insidious and the first 24 hours the diagnosis was indeed quite difficult. А thoracic drain was placed in the cardiosurgery department in another hospital, due to evidence of hydropneumothorax on X-ray with furthermore prolonged stay without recognition of Boerhaave's syndrome. The diagnosis was not recognized promptly in the first six days and was delayed, which could have been fatal for the patient. When obvious evidence of food leakage from the drains appeared, the patient was referred to our thoracic department. A slightly diluted solution of methylene blue was given orally to the patient as this is not standard practice, but a good and reliable method of proving esophageal perforation, especially if a chest tube has been previously placed. The diagnosis was settled with no need for any further imaging investigation, therefore the patient underwent immediate surgery. An immediate operative intervention consisting of bilateral thoracotomies, subtotal esophageal resection, esophagostomy, gastrostomy, debridement, wide opening of both mediastinal pleurae, and early decortication of both lungs was performed. In the presented case, thoracoscopic interventions are not appropriate due to the severe septic state of the patients at admission in our department. Open surgery provides better opportunities for total debridement, decortication, thorough cleaning of the pleural cavity, faster mobilization and extirpation of the thoracic esophagus and is safer for the patient. As the rupture occurred more than 72 hours prior to admission to our department, and due to the rupture's dimensions and the inverted and everted edges, placing a stent, inserting thoracic drains, and performing a feeding jejunostomy without exploring both pleural cavities and the mediastinum would be extremely risky for the patient. The patient's critically severe septic condition did not allow reconstruction of the digestive tract at this stage. In cases with delayed diagnosis, large longitudinal ruptures with everted edges, and the presence of bilateral empyemas and mediastinitis, such as in our case, no attempt to preserve the esophagus is indicated, therefore, the only option is subtotal resection with subsequent reconstruction at another stage. We are aiming for a restoration of the continuity of the digestive tract after the patient’s recovery.

## Conclusions

For the successful treatment of this life-threatening condition, Boerhaave's syndrome, the timing of the correct diagnosis, and the presence or absence of an underlying disease, such as motility disorders or benign and malignant esophageal strictures, are of paramount importance. Successful treatment of this insidious condition requires early diagnosis and prompt intervention. In delayed diagnosis and advanced mediastinitis with bilateral empyema, an aggressive surgical approach is mandatory. With a timely diagnosis, conservative interventions could be tried in order to preserve the integrity of the esophagus. 
